# The associations between cardiometabolic index and mortalities in chronic kidney disease

**DOI:** 10.1097/MD.0000000000049696

**Published:** 2026-07-10

**Authors:** Jie Li, Huixia Cao

**Affiliations:** a Department of Nephrology, People’s Hospital of Zhengzhou University, Henan Provincial People’s Hospital, Henan Provincial Key Laboratory of Kidney Disease and Immunology, Zhengzhou, Henan, China.

**Keywords:** cardiometabolic index, cardiovascular diseases, chronic kidney disease, inflammation, National Health and Nutrition Examination Survey

## Abstract

The incidence of chronic kidney disease (CKD) is on the rise, and the presence of cardiovascular diseases can affect the lifespan of patients with CKD. Among CKD patients, uremic toxins gradually accumulate, leading to chronic inflammation and further promoting the progression of CKD. This study aimed to investigate the relationship between cardiometabolic index (CMI) and all-cause and cardiovascular mortality in patients with CKD using data from the National Health and Nutrition Examination Survey and to analyze the potential mediating roles of inflammatory markers. Multivariate Cox regression models were used to assess the association between CMI and mortality, subgroup analyses were performed to test for heterogeneity, and mediation models were used to quantify the proportion of mediation by inflammatory markers. The statistical software was R 4.3.2, and the significance threshold was set at *P* < .05. Among 3815 CKD patients followed for a median of 99.89 months, 1391 all-cause and 516 cardiovascular deaths occurred. After multivariate adjustment, a higher CMI was associated with increased all-cause mortality (hazard ratio = 1.12, 95% confidence interval = 1.03–1.22), but not with cardiovascular mortality. CMI was positively correlated with leukocyte (β = 0.40) and neutrophil (β = 0.18) counts (all *P* < .001). Mediation analysis showed that leukocytes and neutrophils mediated 5.60% and 7.27% of the effect of CMI on all-cause mortality, respectively (*P* < .01). CMI may serve as a potential biomarker for prognostic assessment in patients with CKD and emphasize the key role of systemic inflammation in metabolic-renal interactions.

## 1. Introduction

Chronic kidney disease (CKD) is a major public health problem worldwide, affecting more than 850 million people.^[1]^ Due to the increased prevalence of diabetes and hypertension, the number of affected individuals is expected to rise.^[2]^ Without timely intervention, kidney disease may progress to kidney failure, leading to severe complications and even death. According to the Global Burden of Disease Study, kidney disease rose from the 19th to the ninth leading cause of death in the world between 2000 and 2021, with deaths increasing by 95%. CKD is projected to become the leading cause of death globally and the fifth leading cause of shortened life expectancy by 2040.^[3,4]^

Metabolic diseases, cardiovascular disease (CVD), and kidney disease are closely intertwined. CKD is more common in people with diabetes, and both CVD and diabetes shorten the lifespan of CKD patients. Therefore, identifying modifiable risk factors, especially those related to cardiometabolic health, is crucial for reducing mortality in this population. In 2023, the American Heart Association introduced a concept called cardiovascular-kidney-metabolic syndrome.^[5]^ Cardiovascular-kidney-metabolic syndrome is usually a multisystemic chronic systemic disease caused by the interaction of multiple factors, with common pathophysiological features including chronic inflammation, insulin resistance, enhanced renin-angiotensin-aldosterone system activity, dyslipidemia, and oxidative stress.^[6]^ Inflammation is strongly linked to CKD. In CKD patients, the accumulation of uremic toxins promotes a state of chronic inflammation, which further drives disease progression.^[7,8]^ Elevated inflammatory markers are associated with higher mortality in CKD populations,^[9,10]^ suggesting that inflammation may mediate long-term outcomes. The cardiometabolic index (CMI) was introduced as a novel anthropometric index by Wakabayashi and Daimon in 2015.^[11]^ It correlates with insulin resistance and diabetes risk,^[12]^ and can be improved by anti-inflammatory diets.^[13]^ However, no study has yet evaluated the association between CMI and mortality specifically in a CKD population.

Therefore, we conducted a study using the National Health and Nutrition Examination Survey (NHANES) database to assess the correlation between CMI and mortality in the CKD population and further examined whether inflammatory markers mediate this relationship.

## 2. Methods

### 2.1. Data source

Data for this study were obtained from the NHANES, which is available on the NHANES website (https://www.cdc.gov/nchs/nhanes/). NHANES is a survey based on data regarding the health status of the US population. The NHANES program has been ongoing since 1999, focusing on a range of issues related to health and nutrition. Approval for the research was granted by the National Center for Health Statistics Research Ethics Review Board under the protocol number (Continuation of Protocol #2011-17), ensuring that all participants provided informed consent.

### 2.2. Study population

**Figure 1.**
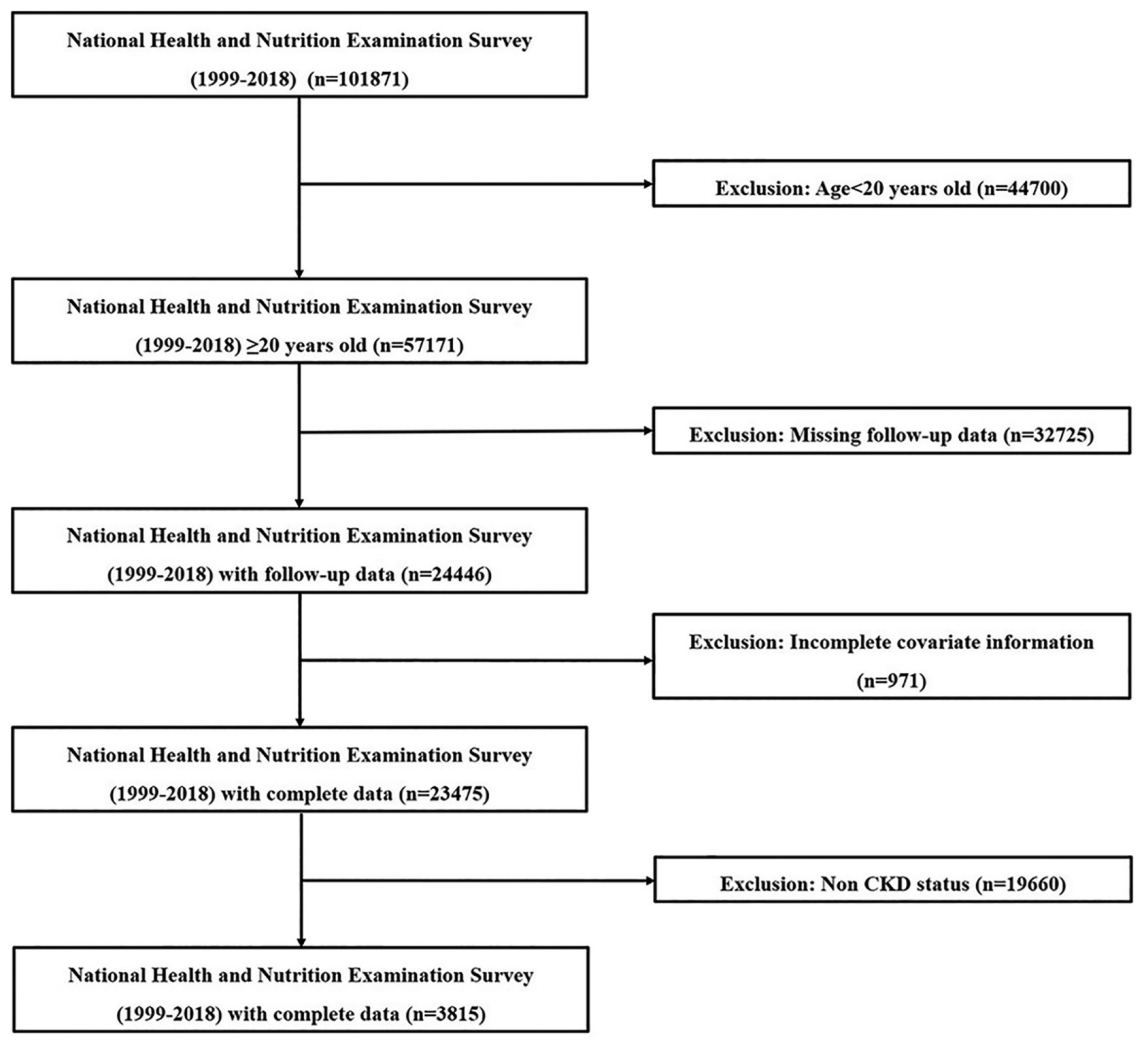
Flowchart of participant selection from NHANES 1999 to 2018. CKD = chronic kidney disease, NHANES = National Health and Nutrition Examination Survey.

In this prospective cohort study, we screened and analyzed data from ten 2-year periods from 1999 to 2018, with a total of 101,871 participants. Initially, participants under 20 years of age (n = 44,700) were excluded. Subsequently, people with no follow-up data were excluded (n = 32,725). Next, people with incomplete covariate information (n = 971) were removed. Finally, people with non-CKD status were excluded (n = 19,660), and a total of 3815 participants were included in the study (Fig. 1).

### 2.3. CKD definition

The estimated glomerular filtration rate (eGFR) and urinary albumin-to-creatinine ratio (ACR) were calculated according to the following equations^[14,15]^:


$$\begin{array}{cc} & eGFR\left(mL/min/1.73\;m^{2}\right)\\ & =141\times min{\left(Scr/\kappa ,\;1\right)}^{\alpha }\times max{\left(Scr/\kappa ,1\right)}^{-1.209}\\ & \times 0.993^{Age}\times 1.018\left(if\;female\right)\times 1.159\;\left(if\;Black\right) \end{array}$$

Notably, Scr indicates serum creatinine concentration (mg/dL), κ is 0.9 for males and 0.7 for females, and α is −0.411 for males and −0.329 for females.


$$\begin{array}{cc} & ACR\left(mg/g\right)\\ & =urine\;albumin\;\left(mg/L\right)/urine\;creatine\;\left(mg/dL\right)\\ & \times 100 \end{array}$$

Based on a comprehensive review of the literature, the definition of CKD is based on the following criteria: eGFR < 60 mL/min/1.73 m^2^ and ACR > 30 mg/g.^[16,17]^

### 2.4. Definition of CMI

The CMI was calculated according to the following formula^[11]^:


$$\begin{array}{cc} CMI & =TG\;\left(mmol/L\right)/HDL-C\;\left(mmol/L\right)\\ & \times WC\left(cm\right)/height\left(cm\right)\; \end{array}$$

TG, triglycerides; HDL-C, high-density lipoprotein cholesterol; WC, waist circumference.

In our study, CMI was treated as a continuous variable, and all enrolled participants were grouped according to the quartiles of CMI values for subsequent analysis.

### 2.5. Outcome assessment

The primary outcomes were all-cause mortality and cardiovascular mortality. Survival data were obtained by linking participants to the National Death Index through December 31, 2019. Cardiovascular mortality was defined using International Statistical Classification of Diseases, 10th Edition, codes I00–I09, I11, I13, I20–I51, or I60–I69.^[18]^

### 2.6. Covariates

In this study, a range of covariates were collected, including socioeconomic characteristics such as sex, age, race, marital status, education level, and family poverty-to-income ratio (PIR). Personal habits and comorbidities included smoking status, alcohol consumption status, diabetes, and hypertension. Examination and laboratory tests included body mass index (BMI), waist circumference, height, waist-to-height ratio (WHtR), leukocyte, neutrophil, lymphocyte, neutrophil-to-lymphocyte ratio (NLR), systemic immune-inflammatory index (SII), platelets, alanine aminotransferase, aspartate aminotransferase, triglycerides, total cholesterol (TC), low-density lipoprotein cholesterol, HDL-C, ACR, and eGFR.

The formula for calculating relevant indicators is as follows:


$$\begin{array}{c} WHtR=WC\;\left(cm\right)/height\;\left(cm\right)\; \end{array}$$

$$\begin{array}{c} NLR=Neutrophil/Lymphocyte\; \end{array}$$

$$\begin{array}{c} SII=Platelet\times Neutrophil/Lymphocyte\; \end{array}$$

### 2.7. Statistical analysis

All data were analyzed using the R project (version 4.3.2; R Foundation for Statistical Computing). Continuous variables were expressed as mean ± standard deviation. Categorical variables were expressed as numbers (percentages, %). A chi-square test was performed for the categorical variables. *P* < .05 was considered statistically significant.

Cox proportional hazards regression models were fitted using the survival package (version 3.8-3; https://CRAN.R-project.org/package=survival) to assess the association between different levels of CMI and mortality in the CKD population.^[19]^ Model 1 was unadjusted. Model 2 adjusted for age, gender, race, marital status, education level, and PIR. Model 3 made additional adjustments for smoking, drinking, hypertension, diabetes, BMI, WHtR, platelets, and TC based on Model 2. In addition, subgroup analyses were conducted to investigate potential differences among specific populations, including gender, race, marital status, education level, PIR, smoking, and drinking subgroups. Multivariate logistic and Cox regression analyses were performed to analyze the relationship between inflammatory indicators such as leukocytes, neutrophils, lymphocytes, NLR, and SII, and CMI and mortality, respectively. Mediation analysis was conducted using the mediation package (version 4.5.0; https://CRAN.R-project.org/package=mediation) to examine whether inflammation-related indicators mediated the aforementioned association.^[20,21]^ The analyses were adjusted for age, sex, race, marital status, education level, PIR, smoking, drinking, hypertension, diabetes mellitus, BMI, WHtR, platelets, and TC.

## 3. Results

### 3.1. Participant characteristics

This study included 3815 individuals with CKD with a mean age of 58.70 ± 18.04 years; 42.81% were male. The mean CMI value was 0.81 ± 0.66. Participants in higher CMI quartiles had higher leukocyte, neutrophil, and lymphocyte counts and also had a higher prevalence of hypertension and diabetes (Table 1).

**Table 1:** Baseline characteristics of the study population according to quartile groups of CMI.

Variable	Total (n = 3815)	Q1 (n = 911)	Q2 (n = 973)	Q3 (n = 994)	Q4 (n = 937)	*P* value
Age, yr						<.001
20–39	505 (18.17)	159 (24.06)	124 (19.21)	101 (12.98)	121 (16.45)	
40–59	826 (28.62)	194 (27.73)	174 (24.04)	220 (29.00)	238 (33.67)	
≥60	2484 (53.21)	558 (48.20)	675 (56.75)	673 (58.02)	578 (49.88)	
Age, yr	58.70 (18.04)	56.04 (19.86)	59.97 (18.61)	61.28 (16.50)	57.49 (16.53)	<.001
Gender, n (%)						<.001
Male	1797 (42.81)	362 (33.34)	432 (35.98)	459 (43.92)	544 (57.97)	
Female	2018 (57.19)	549 (66.66)	541 (64.02)	535 (56.08)	393 (42.03)	
Race, n (%)						<.001
Mexican American	631 (8.60)	86 (5.61)	143 (8.32)	189 (9.96)	213 (10.50)	
Other Hispanic	266 (4.98)	40 (3.65)	63 (4.53)	77 (5.63)	86 (6.12)	
Non-Hispanic White	1551 (61.75)	332 (58.59)	401 (62.05)	386 (59.20)	432 (67.18)	
Non-Hispanic Black	1079 (17.63)	379 (25.23)	306 (19.57)	254 (16.27)	140 (9.44)	
Other race	288 (7.04)	74 (6.93)	60 (5.54)	88 (8.94)	66 (6.76)	
Marital status, n (%)						<.001
Yes	2058 (57.18)	431 (51.27)	503 (53.45)	544 (59.08)	580 (64.89)	
No	1757 (42.82)	480 (48.73)	470 (46.55)	450 (40.92)	357 (35.11)	
Education, n (%)						.032
Below high school	1342 (25.36)	266 (21.17)	328 (25.28)	359 (26.08)	389 (28.92)	
High school or above	2473 (74.64)	645 (78.83)	645 (74.72)	635 (73.92)	548 (71.08)	
Family PIR, n (%)						.767
Poor	775 (15.57)	188 (16.63)	185 (14.60)	193 (14.96)	209 (16.09)	
Not poor	3040 (84.43)	723 (83.37)	788 (85.40)	801 (85.04)	728 (83.91)	
Smoking, n (%)						.423
Yes	696 (19.60)	190 (20.57)	154 (16.69)	177 (19.89)	174 (21.23)	
No	3120 (80.40)	721 (79.43)	819 (83.31)	817 (80.11)	763 (78.77)	
Drinking, n (%)						.015
Yes	2362 (65.78)	583 (68.77)	610 (64.45)	571 (60.72)	598 (69.17)	
No	1453 (34.22)	328 (31.23)	363 (35.55)	423 (39.28)	339 (30.83)	
Hypertension, n (%)						<.001
Yes	2368 (57.21)	496 (45.19)	598 (55.31)	654 (63.93)	620 (64.41)	
No	1447 (42.79)	415 (54.81)	375 (44.69)	340 (36.07)	317 (35.59)	
Diabetes, n (%)						<.001
Yes	1316 (29.43)	172 (13.84)	301 (23.94)	395 (34.96)	448 (44.96)	
No	2499 (70.57)	739 (86.16)	672 (76.06)	599 (65.04)	489 (55.04)	
BMI, kg/m^2^	30.09 (7.64)	25.41 (6.04)	28.72 (6.55)	31.84 (6.97)	34.37 (7.78)	<.001
Waist, cm	102.93 (18.06)	89.72 (14.67)	99.24 (14.30)	107.76 (15.01)	114.98 (17.42)	<.001
Height, cm	166.07 (10.00)	164.88 (9.10)	164.93 (9.60)	165.87 (10.11)	168.58 (10.67)	<.001
WHtR	0.62 (0.10)	0.54 (0.09)	0.60 (0.09)	0.65 (0.09)	0.68 (0.10)	<.001
Leukocyte, 10^9^/L	7.25 (2.82)	6.52 (2.28)	6.96 (3.20)	7.62 (2.78)	7.91 (2.76)	<.001
Neutrophil, 10^9^/L	4.43 (1.94)	4.00 (1.97)	4.24 (1.74)	4.64 (1.67)	4.85 (2.22)	<.001
Lymphocyte, 10^9^/L	1.98 (1.67)	1.76 (0.68)	1.92 (2.54)	2.10 (1.79)	2.16 (0.95)	<.001
NLR	2.55 (1.48)	2.56 (1.71)	2.54 (1.43)	2.56 (1.30)	2.51 (1.44)	.933
SII	629.02 (502.88)	615.35 (487.00)	637.54 (487.83)	642.94 (380.69)	620.15 (626.43)	.658
Platelets, 10^9^/L	247.30 (73.57)	240.09 (71.92)	250.94 (76.28)	251.68 (73.26)	246.52 (72.29)	.119
ALT, U/L	25.27 (47.10)	22.17 (18.78)	22.15 (17.53)	23.67 (14.00)	33.07 (89.10)	.014
AST, U/L	26.32 (18.86)	27.59 (22.75)	24.97 (14.66)	24.30 (9.68)	28.41 (24.20)	.001
TG, mmol/L	1.51 (0.79)	0.75 (0.24)	1.18 (0.29)	1.59 (0.39)	2.52 (0.71)	<.001
TC, mmol/L	4.98 (1.15)	4.88 (1.12)	4.98 (1.14)	4.93 (1.16)	5.15 (1.15)	.002
LDL, mmol/L	2.88 (0.99)	2.65 (0.89)	2.95 (0.99)	2.95 (1.01)	2.96 (1.02)	<.001
HDL, mmol/L	1.42 (0.49)	1.89 (0.59)	1.49 (0.31)	1.25 (0.25)	1.04 (0.22)	<.001
ACR, mg/g	195.30 (681.70)	146.50 (453.98)	177.89 (575.28)	174.19 (596.84)	282.64 (977.87)	.003
eGFR, mL/min/1.73 m^2^	83.53 (32.36)	87.39 (32.79)	82.20 (32.02)	78.20 (31.66)	86.32 (32.19)	<.001
CMI	0.81 (0.66)	0.23 (0.08)	0.48 (0.08)	0.82 (0.13)	1.71 (0.67)	<.001
All-cause mortality						.003
Yes	1391 (31.08)	269 (24.68)	388 (32.66)	373 (33.26)	361 (33.73)	
No	2424 (68.92)	642 (75.32)	585 (67.34)	621 (66.74)	576 (66.27)	
Cardiovascular mortality						.02
Yes	516 (11.19)	98 (7.72)	147 (12.48)	140 (12.24)	131 (12.32)	
No	3229 (88.81)	813 (92.28)	826 (87.52)	854 (87.76)	806 (87.68)	
Follow-up times (mo)	99.89 (61.71)	93.77 (60.53)	102.83 (61.21)	99.30 (63.01)	103.70 (61.68)	.10

ACR = urinary albumin-to-creatinine ratio, ALT = alanine aminotransferase, AST = aspartate aminotransferase, BMI = body mass index, CMI = cardiometabolic index, HDL = high-density lipoprotein, LDL = low-density lipoprotein, NLR = neutrophil-to-lymphocyte ratio, PIR = poverty-to-income ratio, SII = systemic immune-inflammatory index, TG = triglycerides, WHtR = waist-to-height ratio.

### 3.2. Associations of CMI with all-cause and cardiovascular mortality

Over a median follow-up of 99.89 months, 1391 all-cause deaths and 516 cardiovascular deaths occurred. In Model 1 and Model 2, CMI had no association with all-cause and cardiovascular mortality. In Model 3, after adjusting for all disturbing factors, higher CMI was significantly associated with increased all-cause mortality (hazard ratio [HR] = 1.12, 95% confidence interval = 1.03–1.22). However, we found no significant linear trend across CMI quartiles (*P* = .143) and no significant association with cardiovascular mortality (Table 2).

**Table 2:** The associations of CMI with all-cause mortality and cardiovascular mortality in the CKD population.

	HR (95% CI)					
	Model 1		Model 2		Model 3	
	HR (95% CI)	*P* value	HR (95% CI)	*P* value	HR (95% CI)	*P* value
All-cause mortality						
CMI	1.03 (0.95, 1.12)		1.03 (0.94, 1.12)		1.12 (1.03, 1.22)	
Q1	1		1		1	
Q2	1.24 (1.06–1.45)	.006	0.99 (0.85–1.16)	.904	1.08 (0.92–1.26)	.373
Q3	1.16 (0.99–1.36)	.060	0.97 (0.83–1.13)	.681	1.10 (0.93–1.30)	.251
Q4	1.13 (0.96–1.32)	.131	0.97 (0.82–1.14)	.722	1.14 (0.95–1.36)	.151
*P* for trend		.339		.664		.143
Cardiovascular mortality						
CMI	1.03 (0.90, 1.18)		1.07 (0.93, 1.23)		1.14 (0.99, 1.32)	
Q1	1		1		1	
Q2	1.29 (1.00–1.66)	.052	1.04 (0.81–1.35)	.745	1.14 (0.88–1.48)	.336
Q3	1.20 (0.93–1.55)	.168	1.03 (0.79–1.33)	.839	1.16 (0.88–1.53)	.285
Q4	1.12 (0.87–1.46)	.380	1.05 (0.80–1.38)	.733	1.19 (0.88–1.60)	.251
*P* for trend		.623		.792		.281

Model 1 adjusted for none.

Model 2 adjusted for age, gender, race, marital status, education level, and PIR.

Model 3 adjusted for age, gender, race, marital status, education level, PIR, smoking, drinking, hypertension, DM, weight, leukocytes, platelets, and TC.

CI = confidence interval, CKD = chronic kidney disease, CMI = cardiometabolic index, DM = diabetes mellitus, HR = hazard ratio, PIR = poverty-to-income ratio, TC = total cholesterol.

### 3.3. Subgroup analysis

**Figure 2.**
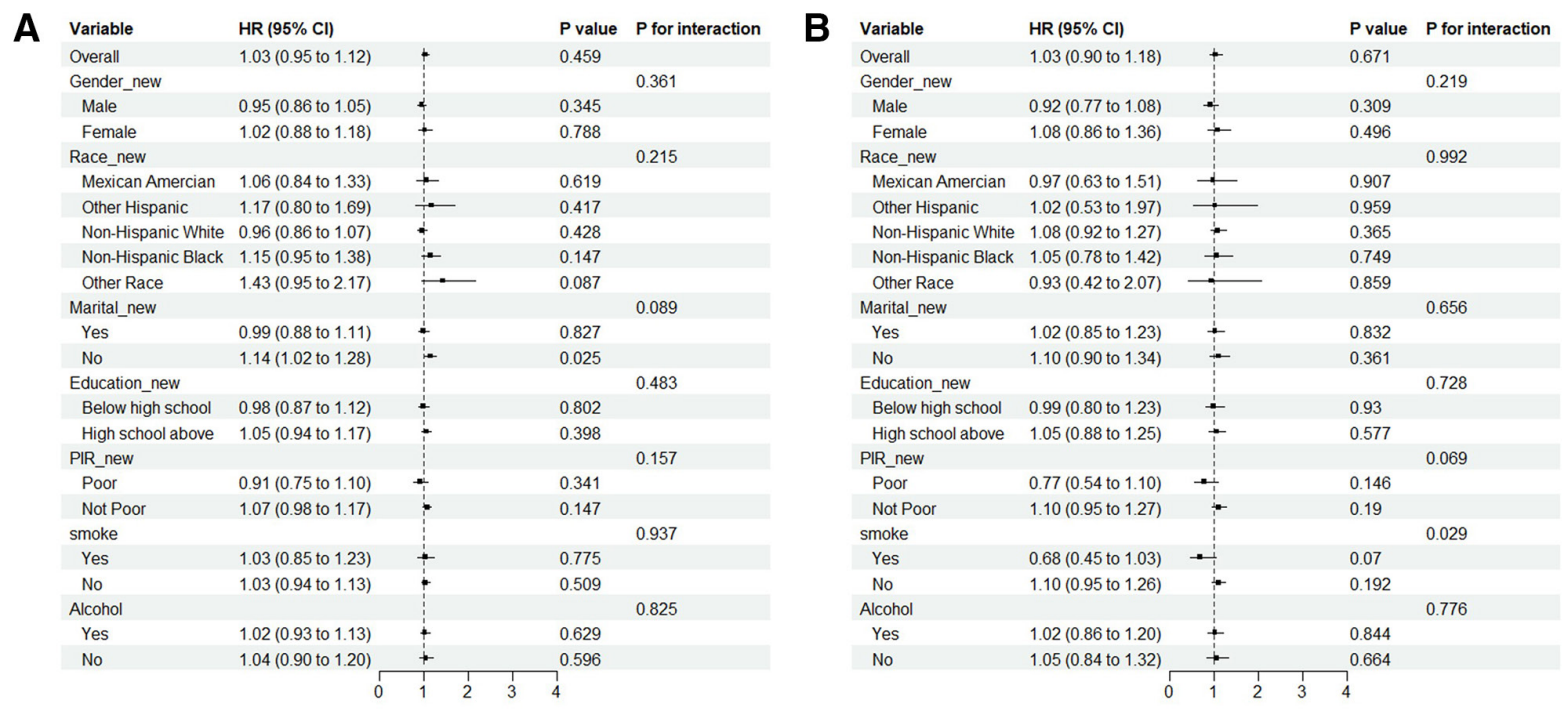
Subgroup analysis and interaction analysis. (A) Subgroup analysis of associations between CMI and all-cause mortality. (B) Subgroup analysis of associations between CMI and cardiovascular mortality. CI = confidence interval, CMI = cardiometabolic index, HR = hazard ratio, PIR = poverty-to-income ratio.

We further performed a subgroup analysis to evaluate the relationships of CMI with all-cause and cardiovascular mortality (Fig. 2). There were no significant differences observed in any subgroup analysis.

### 3.4. Associations of inflammation with CMI and mortality

After adjustment, CMI was positively associated with leukocyte (β = 0.40), neutrophil (β = 0.18), and lymphocyte (β = 0.17) counts and negatively associated with NLR (β = −0.15) and SII (β = −27.18; Table 3). Most inflammatory markers, except lymphocytes, were positively associated with all-cause and cardiovascular mortality in adjusted models (Table 4).

**Table 3:** The associations between CMI and inflammation-related indicators.

	β value	95% CI	*P* value
Leukocyte			
Model 1	0.73	0.57, 0.89	<.001
Model 2	0.63	0.46, 0.80	<.001
Model 3	0.46	0.28, 0.63	<.001
Neutrophil			
Model 1	0.47	0.36, 0.58	<.001
Model 2	0.38	0.27, 0.49	<.001
Model 3	0.23	0.12, 0.34	<.001
Lymphocyte			
Model 1	0.17	0.07, 0.28	.001
Model 2	0.18	0.07, 0.29	.060
Model 3	0.16	0.05, 0.28	.0065
NLR			
Model 1	−0.01	−0.09, 0.06	.720
Model 2	−0.09	−0.17, −0.01	<.001
Model 3	−0.11	−0.19, −0.03	.007674
SII			
Model 1	10.97	−20.58, 42.53	.495
Model 2	0.02	−32.43, 32.48	<.001
Model 3	−20.80	−52.26, 10.66	.19493

Model 1 adjusted for none.

Model 2 adjusted for age, gender, race, marital status, education level, and PIR.

Model 3 adjusted for age, gender, race, marital status, education level, PIR, smoking, drinking, hypertension, DM, weight, platelets, and TC.

CI = confidence interval, CMI = cardiometabolic index, DM = diabetes mellitus, NLR = neutrophil-to-lymphocyte ratio, PIR = poverty-to-income ratio, SII = systemic immune-inflammatory index, TC = total cholesterol.

**Table 4:** The associations of inflammation-related indicators with all-cause mortality and cardiovascular mortality.

	HR (95% CI)		
	Model 1	Model 2	Model 3
All-cause mortality			
Leukocyte	1.031 (1.022, 1.04)	1.024 (1.016, 1.032)	1.02 (1.02, 1.03)
Neutrophil	1.06 (1.05, 1.07)	1.06 (1.05, 1.07)	1.08 (1.07, 1.10)
Lymphocyte	0.94 (0.87, 1.01)	1.01 (0.99, 1.03)	1.01 (0.99, 1.03)
NLR	1.21 (1.19, 1.23)	1.16 (1.13, 1.18)	1.16 (1.13, 1.19)
SII	1.00 (1.00, 1.00)	1.00 (1.00, 1.00)	1.00 (1.00, 1.00)
Cardiovascular mortality			
Leukocyte	1.019 (0.99, 1.04)	1.02 (1.00, 1.04)	1.02 (1.00, 1.03)
Neutrophil	1.05 (1.03, 1.08)	1.06 (1.03, 1.08)	1.09 (1.06, 1.12)
Lymphocyte	0.69 (0.60, 0.80)	0.91 (0.80, 1.03)	0.94 (0.84, 1.06)
NLR	1.20 (1.16, 1.24)	1.15 (1.11, 1.20)	1.14 (1.10, 1.20)
SII	1.00 (1.00, 1.00)	1.00 (1.00, 1.00)	1.00 (1.00, 1.00)

Model 1 adjusted for none.

Model 2 adjusted for age, gender, race, marital status, education level, and PIR.

Model 3 adjusted for age, gender, race, marital status, education level, PIR, smoking, drinking, hypertension, DM, weight, platelets, and TC.

CI = confidence interval, DM = diabetes mellitus, HR = hazard ratio, NLR = neutrophil-to-lymphocyte ratio, PIR = poverty-to-income ratio, SII = systemic immune-inflammatory index, TC = total cholesterol.

### 3.5. Mediating role of inflammation-related indicators

**Figure 3.**
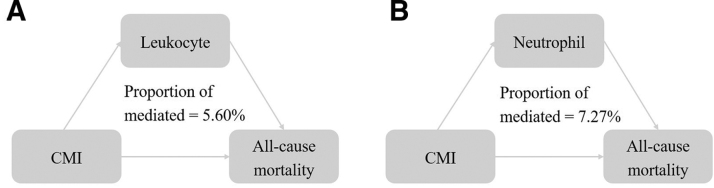
Analysis of the mediation by leukocytes (A) and neutrophils (B) of the associations of CMI with all-cause mortality. CMI = cardiometabolic index.

Mediation analysis revealed that leukocyte and neutrophil counts mediated 5.60% and 7.27% of the association between CMI and all-cause mortality, respectively (*P* < .01). Lymphocyte, NLR, and SII did not show significant mediating effects (Table 5, Fig. 3).

**Table 5:** Analysis of the mediation by inflammation-related indicators of the associations of CMI with all-cause mortality and cardiovascular mortality.

	ACME (indirect effect)		ADE (direct effect)		Total effect		Proportion mediated	
	HR (95% CI)	*P* value	HR (95% CI)	*P* value	HR (95% CI)	*P* value	HR (95% CI)	*P* value
All-cause mortality								
Leukocyte	0.00262 (0.00114, 0.01)	.002**	0.04412 (0.01831, 0.07)	.002**	0.04674 (0.02146, 0.07)	<.001**	0.05597 (0.02141, 0.17)	.002**
Neutrophil	0.00349 (0.00186, 0.01)	<.001**	0.04449 (0.01918, 0.07)	.002**	0.04798 (0.02290, 0.07)	<.001**	0.07270 (0.03342, 0.18)	<.001**
Lymphocyte	−0.000214 (−0.005606, 0.00)	.550	0.048192 (0.022789, 0.07)	<.001**	0.047978 (0.022904, 0.07)	<.001**	−0.004459 (−0.135726, 0.01)	.55
NLR	−0.00470 (−0.00834, 0.00)	.008**	0.05268 (0.02811, 0.08)	<.001**	0.04798 (0.02290, 0.07)	<.001**	−0.09801 (−0.26942, −0.02)	.008**
SII	−0.00164 (−0.00775, 0.00)	.29	0.04961 (0.02565, 0.08)	<.001**	0.04798 (0.02290, 0.07)	<.001**	−0.03411 (−0.23058, 0.02)	.29
Cardiovascular mortality								
Leukocyte	−0.000146 (−0.001375, 0.00)	.854	0.019376 (−0.002287, 0.04)	.072	0.019231 (−0.002073, 0.04)	.074	−0.007584 (−0.164587, 0.25)	.872
Neutrophil	9.84e−04 (−9.62e−05, 0.00)	.082	1.88e−02 (−5.27e−04, 0.04)	.072	1.98e−02 (2.09e−04, 0.04)	.046	4.98e−02 (−2.93e−02, 0.48)	.124
Lymphocyte	−0.000688 (−0.003205, 0.00)	.024 *	0.020453 (0.000916, 0.04)	.040 *	0.019765 (0.000209, 0.04)	.046*	−0.034826 (−0.457443, 0.02)	.070
NLR	−0.001538 (−0.003399, 0.00)	.008**	0.021303 (0.001496, 0.04)	.034*	0.019765 (0.000209, 0.04)	.046*	−0.077805 (−0.726965, 0.00)	.054
SII	−0.000358 (−0.002926, 0.00)	.284	0.020123 (0.000587, 0.04)	.046*	0.019765 (0.000209, 0.04)	.046*	−0.018135 (−0.399624, 0.02)	.322

CMI = cardiometabolic index, NLR = neutrophil-to-lymphocyte ratio, SII = systemic immune-inflammatory index.

* *P* < .05.

** *P* < .01.

## 4. Discussion

This study investigated the association between CMI and mortality in a cohort of 3815 patients with CKD while exploring the potential mediating role of systemic inflammation. The primary findings indicate that higher CMI levels were independently associated with an increased risk of all-cause mortality after adjusting for multiple confounders. Furthermore, systemic inflammation, particularly leukocytes and neutrophils, was found to partially mediate this relationship. These findings emphasize the complex interplay between metabolic dysregulation, inflammation, and adverse outcomes in the CKD population.

CMI, initially proposed as a potential indicator of diabetes mellitus risk, integrates information on central obesity (WHtR) and lipid metabolism (triglycerides/HDL-C ratio). A growing body of evidence has linked elevated CMI to various metabolic disorders, including hypertension, hyperuricemia, nonalcoholic fatty liver disease, and albuminuria.^[22–26]^ Notably, a recent cross-sectional study by Guo et al suggested an association between CMI and the presence of CKD itself.^[27]^ However, prior to our investigation, no study had evaluated the prognostic value of CMI for long-term mortality risk specifically within a CKD population. Our study extends the existing literature by demonstrating that CMI is associated with all-cause mortality in this high-risk group.

In our fully adjusted model, each unit increase in CMI was associated with a 12% higher risk of all-cause mortality (HR = 1.12, 95% confidence interval: 1.03–1.22). This positive correlation aligns with established knowledge linking metabolic abnormalities, such as visceral adiposity and insulin resistance, to poor prognosis in CKD.^[28,29]^ It is noteworthy that the HR, while statistically significant, is modest in magnitude. This suggests that the metabolic risk captured by CMI provides incremental prognostic information beyond traditional risk factors. Conversely, we did not observe a significant linear trend across CMI quartiles (*P* = .143). This absence of a clear gradient might be attributed to a nonlinear relationship, a threshold effect where risk escalates only beyond a certain CMI level, or limited statistical power within quartiles. These possibilities warrant examination in future studies with larger sample sizes.

Interestingly, no significant association was found between CMI and cardiovascular mortality in our CKD cohort. This finding appears contrary to some studies conducted in the general population or in individuals with earlier stages of CKD.^[29]^ This discrepancy may be explained by the unique characteristics of our study population. Patients with advanced CKD, like those in our cohort, face a high burden of non-cardiovascular comorbidities and competing risks, such as infections and sepsis, which are leading causes of death.^[30,31]^ As highlighted by Rai et al, such competing risks can mask associations with cardiovascular-specific mortality.^[32]^ Furthermore, as CKD progresses, the pathophysiology of CVD becomes increasingly complex and is influenced by uremia-specific factors (e.g., renal anemia, mineral bone disease), which might attenuate the relative contribution of metabolic syndrome to cardiovascular outcomes compared to earlier disease stages.^[29,33]^

Our mediation analysis offers insights into a potential pathway linking CMI to mortality. We found that leukocyte and neutrophil counts mediated 5.60% and 7.27% of the association between CMI and all-cause mortality, respectively. This finding suggests that systemic inflammation may act as one intermediary in the metabolic-renal interaction. It proposes a plausible pathophysiological framework: an elevated CMI likely reflects a pro-inflammatory metabolic state characterized by visceral adipose tissue expansion, which secretes cytokines such as interleukin-6 and tumor necrosis factor-alpha, promoting chronic low-grade inflammation.^[34–37]^ This inflammatory milieu, coupled with insulin resistance and dyslipidemia, can exacerbate endothelial dysfunction, oxidative stress, and renal tissue injury, potentially accelerating CKD progression and increasing mortality risk.^[38–46]^ However, it is important to emphasize that these mechanisms are speculative because they are based on our observational data, and the reported mediation proportions are modest. This implies that other unmeasured pathways, such as oxidative stress, endothelial dysfunction, or fibrotic processes, likely play substantial roles and deserve investigation.

Some limitations of this study should be recognized. First, residual confounding from unmeasured variables (e.g., dietary patterns, physical activity) may persist despite extensive adjustment. Second, the observational design precludes causal inference; longitudinal or interventional studies are needed to confirm whether reducing CMI improves prognosis. Third, the use of single-center cohorts and potential selection bias may affect external validity. Finally, the modest mediator proportion (5%–7%) implies that other pathways, such as oxidative stress or endothelial dysfunction, may contribute to mortality risk.

## 5. Conclusion

In conclusion, this study emphasizes that CMI is a prognostic indicator of all-cause mortality in CKD, partially mediated by neutrophilic inflammation. These findings advocate for the comprehensive management of metabolic and inflammatory disorders in CKD care. Future research should validate these associations in multiethnic cohorts, elucidate mechanistic pathways, and evaluate targeted therapies to reduce inflammation in high-risk patients.

## Acknowledgments

We express our sincere gratitude to the editor and reviewers for their detailed and valuable suggestions, which led to invaluable improvements in the design and content of our study. We sincerely thank the participants and staff of NHANES for their valuable contributions, as well as all members who contributed to this work.

## Author contributions

**Data curation:** Jie Li.

**Formal analysis:** Jie Li.

**Methodology:** Jie Li.

**Funding acquisition:** Huixia Cao.

**Validation:** Huixia Cao.

**Writing – original draft:** Jie Li.

**Writing – review & editing:** Huixia Cao.
